# Changes in treatment landscape of relapsed or refractory multiple myeloma and their association with mortality: Insights from German claims database

**DOI:** 10.1111/ejh.13523

**Published:** 2020-11-20

**Authors:** Christof Scheid, Igor W. Blau, Leopold Sellner, Boris A. Ratsch, Edin Basic

**Affiliations:** ^1^ Department of Internal Medicine University Hospital of Cologne Cologne Germany; ^2^ Charité University Medicine Berlin Berlin Germany; ^3^ Takeda Pharma Vertrieb GmbH & Co. KG Berlin Germany

**Keywords:** doublets, observational study, relapsed or refractory multiple myeloma, risk reduction, triplets

## Abstract

**Objectives:**

Emerging treatments for relapsed or refractory multiple myeloma (rrMM) have led to increasing options for many patients. This study aimed to assess changes in utilization of these options in Germany with a focus on modern triplet regimens including new agents, such as carfilzomib, ixazomib, elotuzumab and daratumumab, and to evaluate whether this had an impact on rrMM‐related outcomes over time.

**Methods:**

The study population consisted of 1255 rrMM patients who were assigned to one of the following 6 treatment groups: immunomodulatory drug (IMiD)‐based doublets, proteasome inhibitor (PI)‐based doublets, daratumumab monotherapy, PI‐IMiD‐based triplets, monoclonal antibodies (mAbs)‐based triplets, or other treatment.

**Results:**

Use of triplet‐based therapy regimens increased from 5.9% in 2014 to 31.4% in 2017. In parallel, use of IMiD‐based doublets decreased from 74.3% in 2014 to 37.6% in 2017. Over the same time period, the risk of death decreased by 32% and the risk of hospitalization which was reduced by 30%. The risk for serious adverse events remained unchanged.

**Conclusions:**

Between 2014 and 2017, the use of triplet‐based therapy regimens for rrMM in Germany has significantly increased and this was associated with a significant decline in deaths and hospitalizations without an increased incidence of serious adverse events.

**Novelty Statements**

What is the new ASPECT of your work?: Assessment of whether changes in treatment patterns have resulted in improved clinical outcomes of relapsed or refractory multiple myeloma patients in Germany. What is the CENTRAL finding of your work?: Between 2014 and 2017, the use of new triplet‐based therapy regimens for relapsed or refractory multiple myeloma in Germany has significantly increased and this was associated with a significant decline in deaths and hospitalizations without an increased incidence of serious adverse events. What is (or could be) the SPECIFIC clinical relevance of your work?: Increased use of new triplet‐based therapies for treatment of relapsed or refractory multiple myeloma.

## 1 INTRODUCTION

The immunomodulatory drug (IMiD) lenalidomide and the proteasome inhibitor (PI) bortezomib have been the mainstay in the treatment of patients with relapsed or refractory multiple myeloma (rrMM), mostly as doublets in combination with dexamethasone over the last 10‐15 years. More recently, several new agents, such as the PIs carfilzomib and ixazomib, the IMiD pomalidomide and two monoclonal antibodies (mAbs), elotuzumab and daratumumab, have been approved for the treatment of rrMM patients. These new agents, given mostly in triplet combination, have demonstrated to be efficacious in extending progression‐free survival (PFS) and time to progression, and expanded the standard options in treatment of rrMM.^1^, ^2^, ^3^, ^4^, ^5^, ^6^ Several studies have shown that these new agents are being increasingly used for management of rrMM.^7^, ^8^ It is currently not known if these changes also apply for rrMM patients treated in Germany; moreover, it remains to be determined whether the increased use of triplet‐based therapy regimens including these new agents has resulted in improved clinical outcomes. The present retrospective observational study therefore aimed at first, to assess changes in treatment of rrMM patients after the availability of new agents in Germany between 2014 and 2017; and second, to evaluate whether the adoption of these new agents has resulted in changes in rrMM‐related outcomes over time.

## 2 METHODS

### 2.1 Study design and data source

This retrospective observational study was based on the Institute for Applied Health Research (formerly Health Risk Institute, Berlin) database which is an anonymized healthcare claims database with longitudinal data from approximately 7 million Germans insured in one of approximately 70 German statutory health insurances. Previous analyses revealed that the database from the Institute for Applied Health Research has a good external validity to the German population in terms of morbidity, mortality, and drug use.^9^ In brief, the database includes patient demographics, enrollment history, and medical and pharmacy claims. Medical claims include information on outpatient healthcare services and data related to hospital treatment, including admission and discharge dates, diagnoses, operations, and interventions. All diagnoses in the database were coded according to the German modification of the International Classification of Diseases. Pharmacy claims include various information on all outpatient prescriptions dispensed in German pharmacies such as dispensing/prescription date and number of tablets dispensed. Prescription handouts were coded according to the Anatomical Therapeutic Chemical Classification System.

Patient‐level data can be arrayed chronologically to provide a detailed longitudinal profile of all medical and pharmacy services used by each insured member. All patient identifiers were fully encrypted from the database which is therefore compliant with the German data protection regulations. As no patient contact was made and patient information was de‐identified, Institutional Review Board approval was not required.

### 2.2 Study population

**Figure 1:**
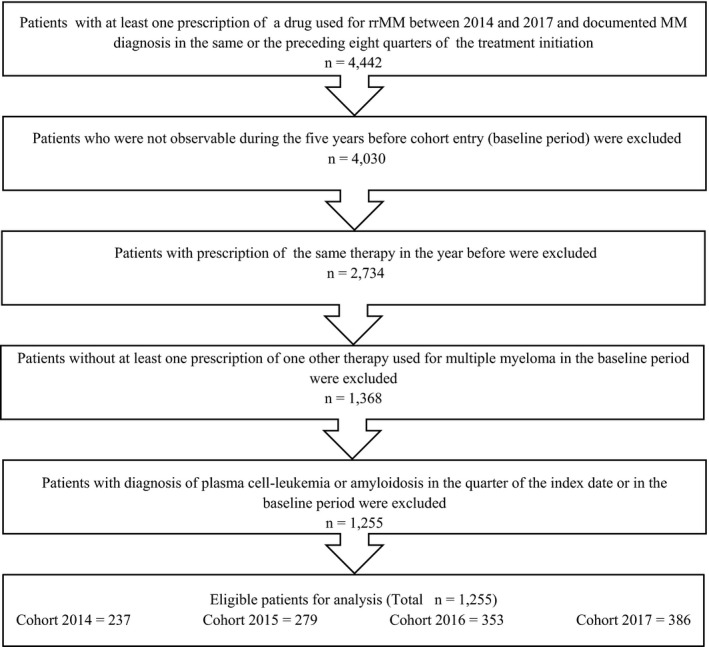
Selection process of the included relapsed or refractory multiple myeloma patients

The study population consisted of all patients who had at least one new prescription of a therapy regimen used for rrMM between January 1, 2014, and December 31, 2017. To obtain a new starter cohort, patients with a prescription of the newly initiated therapy regimen within the previous 12 months were excluded. The index date was defined as the start date of the therapy, and the index quarter as a quarter in which the therapy was prescribed. To focus on relapsed or refractory multiple myeloma, several inclusion and exclusion criteria were applied. Patients without at least one prescription of any other therapy regimen used for multiple myeloma in the last five years were excluded. Furthermore, patients with amyloidosis or plasma‐cell leukemia were excluded. In addition, patients were required to have continuous enrollment in the database for 5 years prior to the index quarter. Derivation of the study population is shown in Figure 1.

### 2.3 Therapy regimens and study outcomes

Based on their prescribed treatment, patients were assigned to one of the following 6 treatment groups: IMiD‐based doublets, PI‐based doublets, Daratumumab Monotherapy (without dexamethasone), PI‐IMiD‐based triplets, mAbs‐based triplets, or other. The composition of each treatment group is provided in Table S1.

The first objective of the study was to assess the proportion of German rrMM patients who did receive one of the above listed therapies. This was evaluated over the entire study period and for each of the 4 sequential annual cohorts separately (years 2014‐2017).

The second objective was to identify factors associated with use of triplet‐based therapies.

The third objective was to determine whether changes in treatment patterns over time resulted in changes in clinical outcomes including death, time to next therapy, febrile neutropenia, pneumonia, thrombosis, number of platelet or red cell transfusions, and number of hospitalization or hospital days (see Table S2 in Supporting Information for exact definitions of the outcomes). In particular, to assess the impact of the introduction of new agents, such as carfilzomib, ixazomib, elotuzumab, and daratumumab, in Germany on the incidence of clinical outcomes, these were evaluated before and after the introduction of the new agents, that is, in rrMM patients identified in 2014 and 2017, respectively; all patients were followed for one year after the index date.

### 2.4 Statistical analysis

Baseline characteristics of the study population were reported as percentages or means ± standard deviation (SD) and were examined during 8 quarters prior to index quarter. Only previous stem cell transplantation (SCT) and number of prior therapies were assessed through the entire baseline period of 5 years. Treatment patterns were descriptively analyzed for each of the 4 annual cohorts and represented separately for the entire study period and by cohort. Additionally, the association between patient characteristics and the use of triplet‐based therapy (PI‐IMiD‐based triplet or mAb‐based triplet) vs doublet‐based therapy (IMiD‐based doublet or PI‐based doublet) was examined using a binary logistic regression model. For this analysis, all doublet‐based therapy regimens and all triplet‐based therapy regimens were grouped into one group, respectively. Daratumumab monotherapy and treatments clustered in the other group were not considered for this analysis. Odds ratios (ORs) were reported for each risk factor, together with their 95% confidence intervals (CIs).

Clinical outcomes before and after the adoption of new agents were compared using Cox proportional hazard models and Poisson regression models with a log link (see Statistical analysis section in Supporting Information for more information). In order to account for possible differences in patients characteristics in 2014 and 2017 cohorts, models were adjusted for age, gender, history of anemia, hypercalcemia, bone lesion, kidney disease, diabetes, cardiovascular disease, prior SCT, and number of previous therapies. Adjusted hazard ratios (HRs) and adjusted rate ratios (RRs) were reported with their 95% CIs.

In addition, unadjusted event rates were estimated in both cohorts and for each of the specified clinical outcomes and were expressed per person‐year.

As a prespecified subgroup analysis, outcome analyses were performed for patients in different age groups (≤70, 71‐75, ≥76).

Data analysis was carried out by the Institute for Applied Health Research, Berlin. A two‐sided *P*‐value < .05 was considered statistically significant.

## 3 RESULTS

### 3.1 Patient population

The overall study population for addressing the first study objective comprised 1255 rrMM patients enrolled in four sequential cohorts between 2014 and 2017. Overall, 70.0% of patients (n = 878) received a doublet‐based therapy regimen, 18.6% (n = 234) received a triplet‐based therapy regimen while 4.7% (n = 59) were treated with daratumumab as monotherapy (Table 1). The remaining 6.7% (n = 84) received a variety of regimens not belonging to the categories above and were thus included in the category “other.” Among 878 patients treated with doublet‐based therapy regimens, 682 (77.7%) were treated with IMiD‐based doublets while 196 (22.3%) were prescribed PI‐based doublets. Similarly, among 234 patients who received a triplet‐based therapy regimens, 176 (75.2%) were treated with PI‐IMiD‐based triplets while 58 (24.8%) received a mAb‐based triplet. Patients who were treated with triplets were on average younger and had less comorbidities than those treated with doublets or daratumumab as monotherapy. Among patients treated with doublet‐based therapy regimens, IMiD‐based doublets were more often used in patients with prior SCT, female patients, and patients who had lower number of prior treatments, compared with patients receiving PI‐based doublets.

**Table 1:** Baseline characteristics of the overall study population at the time of therapy for relapsed or refractory multiple myeloma

Characteristic	IMiD‐based doublet, n = 682	PI‐based doublet, n = 196	Daratumumab Monotherapy, n = 59	PI‐IMiD‐based triplet, n = 176	mAb‐based triplet, n = 58	Other, n = 84
Patient demographics						
Age (mean ± SD)	69.6 (10.4)	69.6 (10.3)	69.5 (9.2)	64.4 (10.8)	67.7 (9.3)	67.1 (11.3)
Age groups (%)						
≤70 y	50.2	14.2	4.3	18.5	5.9	7.0
71‐75 y	53.4	17.5	6.8	12.0	4.0	6.4
≥76 y	62.1	16.7	4.0	7.8	3.0	6.5
Male (%)	61.4	66.8	66.1	60.8	65.5	52.4
Index year (%)						
2014	74.3	14.8	0.0	5.9	0.0	5.1
2015	74.2	9.7	0.0	5.4	0.0	10.8
2016	43.6	19.0	5.9	19.8	4.0	7.6
2017	37.6	17.4	9.8	19.9	11.4	3.9
Medical history						
Charlson Comorbidity Index (mean ± SD)	6.3 (3.0)	6.7 (2.7)	6.4 (3.1)	5.9 (2.7)	5.5 (2.4)	5.6 (2.8)
Number of previous therapies (mean ± SD)	1.4 (0.6)	1.8 (0.8)	2.6 (1.0)	1.5 (0.9)	1.9 (1.1)	1.9 (0.9)
Previous SCT (%)	27.6	32.7	23.7	33.5	40.8	31.0
Anemia (%)	66.7	66.8	74.6	61.4	50.0	58.3
Hypercalcemia (%)	20.2	21.6	27.1	20.4	13.8	21.4
Bone lesion (%)	91.1	94.9	93.2	92.6	91.4	90.5
Diabetes mellitus (%)	26.8	28.1	28.8	22.7	27.6	25.0
Renal insufficiency (%)	39.7	45.9	52.5	36.4	24.1	41.7
Cardiovascular disease (%)	38.6	40.3	40.7	27.3	27.6	29.8

Abbreviations: IMiD, immunomodulatory drugs; mAbs monoclonal antibodies; bone lesion defined as spinal cord compression, surgery to the bone, pathologic fracture or radiation; cardiovascular disease defined as acute myocardial infarction, coronary heart disease, or heart failure; PI, proteasome inhibitor; SCT, stem cell transplantation; SD, standard deviation.

The study population to address the third study objective, treatment outcome in relation to therapy patterns, comprised 623 rrMM patients who were identified in the years 2014 (n = 237) and 2017 (n = 386), respectively. The distribution of demographic and clinical characteristics is depicted in Table 2.

**Table 2:** Baseline characteristics of the study population in cohorts 2014 and 2017

Characteristic	Cohort 2014, n = 237	Cohort 2017, n = 386
Age (mean ± SD)	67.8 (10.3)	68.5 (10.9)
Age groups (%)		
≤70 y	53.2	52.3
71‐75 y	21.1	17.6
≥76 y	25.7	30.1
Male (%)	64.6	62.4
Charlson Comorbidity Index (mean ± SD)	6.2 (3.1)	6.3 (2.8)
Number of previous therapies (mean ± SD)	1.5 (0.6)	1.7 (1.0)
Previous SCT (%)	32.1	30.1
Anemia (%)	67.9	62.7
Hypercalcemia (%)	19.8	18.6
Bone lesion (%)	88.2	93.0
Diabetes mellitus (%)	28.7	25.9
Renal insufficiency (%)	35.4	40.4
Cardiovascular disease (%)	33.3	37.6

Abbreviations: SCT, stem cell transplantation; SD, standard deviation.

### 3.2 Temporal trends in therapy regimens for rrMM

**Figure 2:**
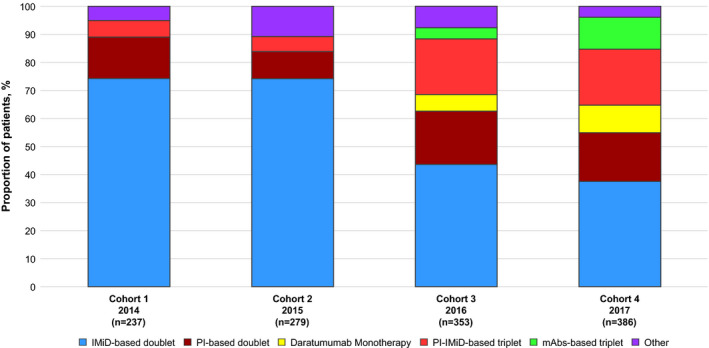
Treatment patterns among patients with relapsed or refractory multiple myeloma between 2014 and 2017

Figure 2 shows the prescribing patterns in the 4 sequential rrMM patient cohorts. Use of PI‐IMiD‐based triplet therapy regimens increased from 5.9% in 2014 to 20.0% in 2017. This change was driven by the increased use of carfilzomib‐ and ixazomib‐based combinations (see Table S1 in Supporting Information for proportions of single therapy regimens). Similar increase in use was also observed for mAb‐based triplets, mainly related to increased use of daratumumab‐ and elotuzumab‐based combinations. The use of daratumumab as monotherapy increased from 6.0% in 2016 (year of approval) to 9.8% in 2017. The small increase in use of PI‐based doublets from 14.8% in 2014 to 17.4% in 2017 was predominantly due to increased use of carfilzomib‐based doublets. In parallel, use of IMiD‐based doublets decreased from 74.3% in 2014 to 37.6% in 2017.

### 3.3 Factors associated with use of triplet‐based therapy

**Figure 3:**
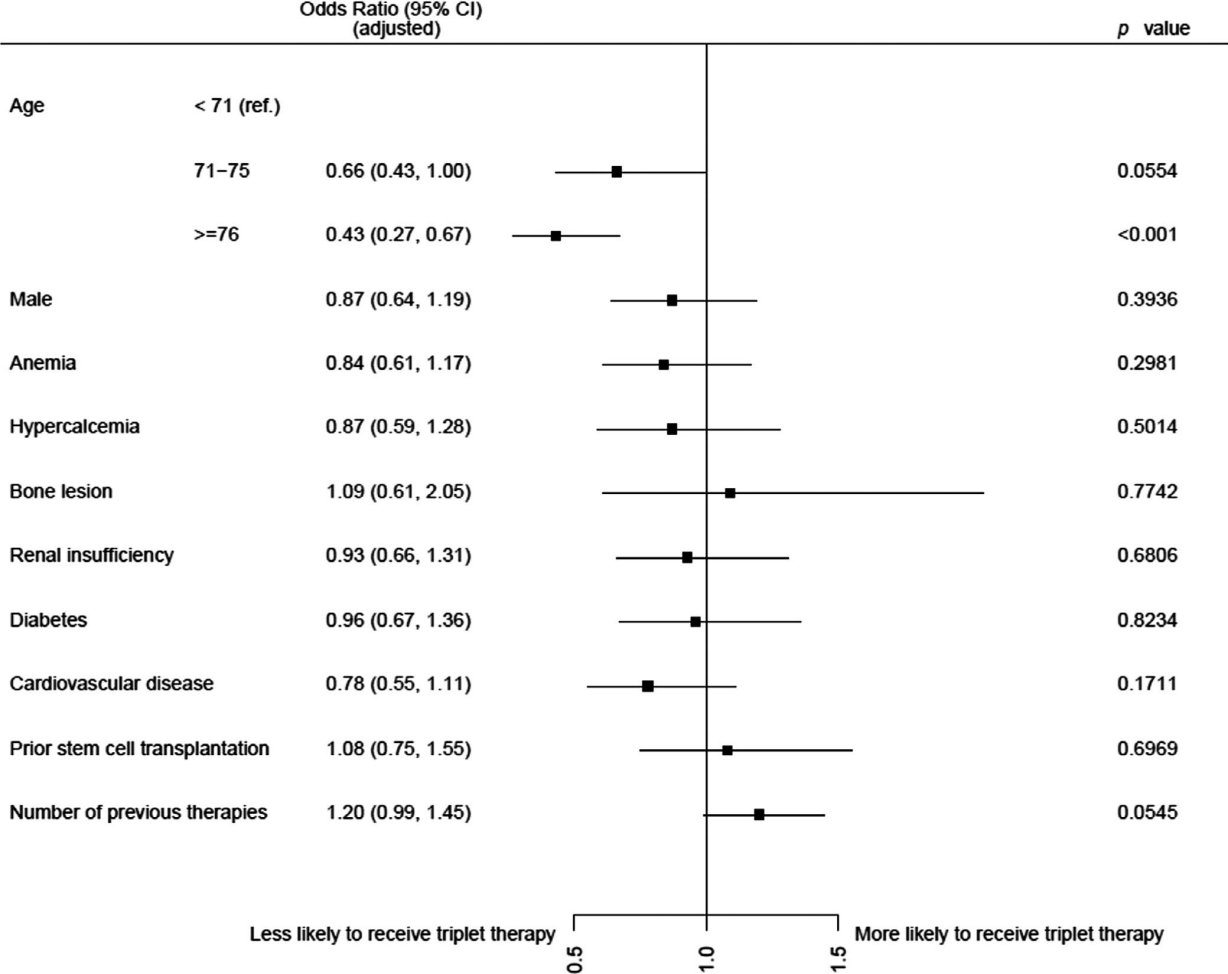
Odds ratios with 95% confidence intervals associated with use of triplet‐based therapies (PI‐IMiD‐based triplet or mAb‐based triplet) vs use of doublet‐based therapies (IMiD‐based doublet or PI‐based doublet), according to age, gender, number of previous therapies, prior stem cell transplantation, and comorbidity

Figure 3 shows associations between patient characteristics available for this analysis and use of triplet‐based (PI‐IMiD‐based triplet or mAb‐based triplet) vs doublet‐based (IMiD‐based doublet or PI‐based doublet) therapy regimens.

Patients aged above 70 were more likely to receive a doublet‐based therapy regimen, while patients at age 70 or below were more often treated with triplet‐based therapy regimens. There was a trend that patients in later lines of therapy received triplet‐based therapy regimens more frequently than doublet‐based therapy regimens.

### 3.4 Changes in treatment landscape over time and their association with clinical outcomes

**Figure 4:**
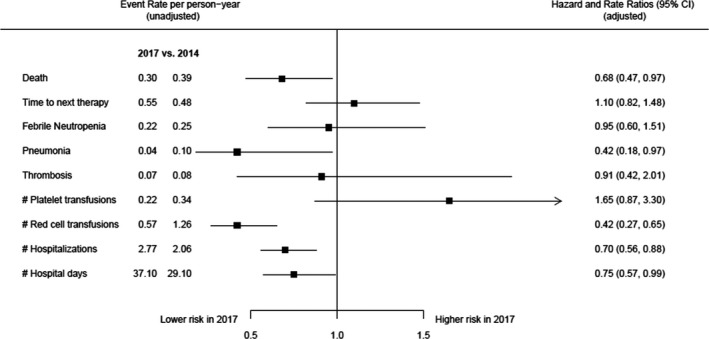
Unadjusted event rates (per person‐year) and adjusted hazard and rate ratios with 95% confidence intervals for comparison of cohorts 2014 and 2017. Adjustment was made for age, gender, number of previous therapies, prior stem cell transplantation, and comorbidity. Abbreviations: #, number; CI, confidence interval

Figure 4 displays the unadjusted event rates, adjusted hazard and rate ratios, and the corresponding forest plot for time to event and count outcomes for comparison of cohorts 2014 and 2017. For cohort 2017, event rates per person‐year of all time to event outcomes except time to next therapy were lower than that for cohort 2014. After adjusting for patient baseline characteristics, patients treated in 2017 had lower risks of death (HR: 0.68, 95% CI: 0.47‐0.97) and pneumonia (HR: 0.42, 95% CI: 0.18‐0.97) compared to patients treated in 2014. The risk of treatment change, febrile neutropenia, and thrombosis was similar between patients treated in 2014 and 2017. A similar pattern was observed for count outcomes. The unadjusted event rates per person‐year of all count outcomes but the number of platelet transfusions was lower in cohort 2017 compared to cohort 2014. The adjustment for patient baseline characteristics resulted in significantly lower risks for red cell transfusions (RR: 0.42, 95% CI: 0.27‐0.65), hospitalizations (RR: 0.70, 95% CI: 0.56‐0.88), and number of hospital days (RR: 0.75, 95% CI: 0.57‐0.99) in cohort 2017 compared to cohort 2014 while the risk of platelet transfusions was similar between the two cohorts.

### 3.5 Outcome risk according to age

Results from subgroup analysis revealed that the benefit with respect to time to event outcome death and count outcomes number of hospitalizations, number of red cell transfusions, and number of hospital days was consistent in all prespecified age subgroups: No significant interaction was observed between these subgroups and cohort membership (Table A1 in Appendix). For the outcomes pneumonia, thrombosis, and platelet transfusions, the number of events was too low to perform a valid comparison between cohorts in the prespecified age subgroups.

## 4 DISCUSSION

### 4.1 Main findings

Our study reveals several important findings: (a) In a large unselected population of rrMM patients in Germany, the use of PI‐IMiD‐based and mAb‐based triplet therapy regimens significantly increased between 2014 and 2017, mainly due to more frequent prescription of combinations including new agents, such as carfilzomib, ixazomib, elotuzumab, and daratumumab; (b) the use of IMiD‐based doublet therapy regimens decreased by about half between 2014 and 2017, however, approximately 38% of rrMM patients were still receiving this therapy; (c) in parallel with the increased prescription of triplet‐based therapy regimens, there was a significant decline in death and hospitalization rates without an increase of typical adverse events, such as febrile neutropenia, thrombosis, or platelet transfusions; (d) the improvement in outcomes observed between 2014 and 2017 in the overall population was maintained across age subgroups.

### 4.2 Use of triplet‐based therapy regimens in rrMM over time

Our findings based on data collected over 4 sequential years demonstrate a considerable increase in treated rrMM patients, likely due to improvements in survival in multiple myeloma and increased number of different treatment options and regimens for rrMM, allowing for more lines of therapy with varying regimens.^10^, ^11^, ^12^, ^13^, ^14^ Over the same time period, a significant increase in the rate of triplet‐based therapy regimens was observed. This change was predominantly due to increased use of new agents, such as carfilzomib, ixazomib, elotuzumab, and daratumumab, a finding which is in line with other observations^7^, ^8^ and reflects the increase in approved drug combinations for the treatment of myeloma. Of note, the increase in triplet‐based therapy regimen use exceeded the decrease in doublet‐based therapy regimens use suggesting that the introduction of new agents is likely to have contributed to improved overall treatment rates among rrMM patients. These changes are also in agreement with the most recent guidelines which prioritize triplet‐based therapy regimens over other forms of treatment for rrMM, at least at first relapse.^15^ However, the present analysis also shows that the use of doublet‐based therapy regimens among rrMM patients in Germany is still common, especially in elderly patients. This might be related to the lack of evidence regarding the benefit of triplets as this group of patients is often underrepresented in clinical trials.^16^, ^17^ In addition, frail elderly patients are more susceptible to drug‐related toxicities, particularly with regard to hematologic toxicity limiting widespread use of more intensive triplet‐based therapy regimens. This becomes particularly evident by the increased use of daratumumab monotherapy in such a group of patients. On average, patients treated with daratumumab monotherapy were of similar age and had even more comorbidities including anemia, renal failure, and history of cardiovascular diseases than patients receiving doublet‐based therapy regimens.

### 4.3 Factors associated with use of triplet‐based therapy regimens

Findings from the binary logistic model evaluating factors associated with use of triplet‐based therapy regimens revealed that age is the main driver for the choice of therapy among rrMM patients. Younger patients were more likely to receive more intensive triplet‐based therapy regimens than older patients. This may indicate the hesitancy and perception of increased toxicity risk among physicians when considering more intensive regimens in an older patient population that may be more frail and have a higher comorbidity burden. Furthermore, the goal of therapy is different in these groups: for young, fit patients the goal might be to achieve a complete remission and improve survival, while in elderly, frail patients it might be more important to improve and maintain the quality of life.^18^, ^19^ Patients with more prior treatments were more likely to receive triplet‐based therapy as compared to doublet‐based therapy. This may be explained by the need to introduce new therapy combinations in patients who already have had the same drug as part of doublet‐ or triplet‐based therapies.

### 4.4 Association of increased triplet‐based therapy regimens use and clinical outcomes

In pivotal phase III trials, new agents, given mostly as triplet combinations, have significantly reduced the risk of disease progression or death.^1^, ^2^, ^3^, ^4^, ^5^ The risk reduction ranged from 63% to 26% and was achieved within relatively short follow‐up times of 13‐24 months. However, for carfilzomib and elotuzumab in combination with lenalidomide and dexamethasone, the benefit regarding the improvements in overall survival (OS) alone was observed after a much longer follow‐up of at least 4 years and for other triplet combinations the OS data are still immature and not yet available.^20^, ^21^ To date, real‐world data studies of changes in rrMM‐related outcomes over time are very scarce. For instance, Kumar et al^22^ reported a marked improvement of OS in MM generally and in rrMM specifically from the year 2000 onward. Another study utilizing a large US electronic medical records database of adult patients with rrMM found that the use of new treatments has contributed to both longer OS and time to next treatment.^7^

The present study indicated that an increase in prescription of triplet‐based therapy regimens was associated with a significant decline in the risk of death amounting to an overall 32% reduction in death between 2014 and 2017. While it is not possible to attribute, this change in practice to a single cause, increased use of triplet‐based therapy regimens, guideline changes, and improvements in supportive care strategies may have been contributory. Furthermore, the availability of new agents and thus much broader treatment repertoire and strategies allows timely and more efficacious interventions at each relapse phases. This is further supported by the fact that time to next treatment did not differ between 2014 and 2017. Of note, the time to next treatment was defined as time from therapy initiation until prescription of new therapy during follow‐up. Thus, it appears that the improved survival was not directly correlated with the duration of response of a given line of treatment but rather a better chance to receive a subsequent line of therapy.

Furthermore, it is important to note that the OS benefit was achieved within a shorter time frame than in the respective phase III trials of the new agents. Although the studies are not directly comparable due to different study objectives, some other factors may have been responsible for the observed discrepancies regarding the OS improvements, such as differences in patient populations, timing of rrMM therapy initiation or physician and patient preferences. For example, patients in daily practice tend to be older, receive more prior lines of therapy and have more comorbidities including renal disease, cardiovascular disease, anemia, and hypercalcemia than in pivotal trials; hence, patients in routine clinical practice may have lower survival rates compared to those in pivotal trials.^17^, ^23^ Stratification for subgroups by age demonstrated that the reduction in death rates was consistent throughout age groups indicating that also patients with older age benefitted from the more widespread use of triplet‐based therapy regimens. On the other hand, an increased use of more intensive triplet‐based therapy regimens might be expected to come at a price of a higher rate of adverse events. However, no increase in risk for serious adverse events was observed between 2014 and 2017. In fact, the risk for hospitalizations and red cell transfusions was significantly lower for patients treated in 2017 as compared to 2014.

Furthermore, it is important to emphasize that in Germany, all medical costs related to cancer care are covered in full by the German healthcare system. In principle, there is no substantial out‐of‐pocket cost that may prevent patients from accessing cancer care. Therefore, this study may contribute to better understanding of the “real‐world” impact of new therapies outside of clinical studies when all patients have equal access to available innovative treatments.

### 4.5 Limitations of the study

Our study has several limitations inherent to any study using claims data. The claims database lacks relevant information on clinical data, including clinical stage of disease, information on cytogenetic abnormalities, and laboratory data on renal function. Another concern may be the potential for coding errors inherent to any retrospective analysis of claims databases. Furthermore, the population covered by the database used in this study may not be generalizable beyond the statutory health insured population in Germany, for example persons with private health insurance. However, the majority (ca. 85%) of the population in Germany is insured in the statutory health insurance. Though the analyses are adjusted for patient baseline demographic and clinical characteristics, unmeasured confounding may still be present. At the time of analysis, data were available until 2018. Thus, the follow‐up for the comparison of the clinical outcomes was limited to one year. Finally, our study describes only an association between therapy changes over time and clinical outcomes but cannot prove any causality.

## 5 CONCLUSION

Our observations in a large cohort of rrMM patients treated in Germany demonstrate an increased use of triplet‐based therapy regimens in recent years, predominantly due to the more widespread use of regimens including new agents, such as carfilzomib, ixazomib, elotuzumab, and daratumumab. This was associated with a significant decline in death and hospitalization rates without an increased risk for serious adverse events.

## 6 ACKNOWLEDGEMENTS

The Open Access funding was provided by Takeda Pharma Vertrieb GmbH & Co. KG.

## Supporting information

Supplementary Material

## Data Availability

Data openly available in a public repository that issues datasets with DOIs.

## 1

**Table A1:** Adjusted hazard ratios and rate ratios with their 95% confidence intervals (CI) and *P*‐values for interactions of time to event and count outcomes for the age subgroups for comparison of cohorts 2014 and 2017

Outcomes	Hazard ratio (95% CI) (adjusted)	*P*‐value for interaction
Death		
≤70 y	0.58 (0.31‐1.10)	.8340
71‐75 y	0.61 (0.29‐1.26)	
≥76 y	0.83 (0.46‐1.50)	
Time to next therapy		
≤70 y	1.04 (0.70‐1.53)	.5039
71‐75 y	0.82 (0.42‐1.61)	
≥76 y	1.41 (0.69‐2.87)	
Febrile neutropenia		
≤70 y	(0.49‐1.39)	.5437
71‐75 y	(0.33‐3.56)	
≥76 y	1.92 (0.21‐17.40)	
Rate ratio (95% CI) (adjusted)		
Number of red cell transfusions		
≤70 y	0.37 (0.20‐0.68)	.8602
71‐75 y	0.42 (0.15‐1.06)	
≥76 y	0.46 (0.17‐1.24)	
Number of hospitalizations		
≤70 y	0.62 (0.45‐0.85)	.5665
71‐75 y	0.85 (0.50‐1.44)	
≥76 y	0.70 (0.44‐1.12)	
Number of hospital days		
≤70 y	0.61 (0.41‐0.90)	.3328
71‐75 y	0.87 (0.50‐1.50)	
≥76 y	0.90 (0.52‐1.59)	
