# Supplementary material for: Changes in treatment landscape of relapsed or refractory multiple myeloma and their association with mortality: Insights from German claims database
Source: Eur J Haematol. 2020 Nov 20;106(2):148–57. doi: 10.1111/ejh.13523 (PMC7894176; doi:10.1111/ejh.13523)
Supplement: Supplementary file 1 — Supplementary Material [file EJH-106-148-s001.docx]

Table S1: Distribution of all relapsed refractory therapy regimens between 2014 and 2017

| **Treatment group** | **Specific combination** | **2014**  **n (%)** | **2015**  **n (%)** | **2016**  **n (%)** | **2017**  **n (%)** |
| --- | --- | --- | --- | --- | --- |
| IMiD-based doublets | Overall | 176 (74) | 207 (74) | 154 (44) | 145 (38) |
|  | Lenalidomide + Dexamethasone | 121 (51) | 150 (54) | 118 (33) | 118 (31) |
|  | Pomalidomide + Dexamethasone | 49 (21) | 48 (17) | 31 (9) | 26 (7) |
|  | Thalidomide + Dexamethasone | 6 (2) | 9 (3) | 5 (1) | <5 (/) |
| PI-based doublets | Overall | 35 (15) | 27 (10) | 67 (19) | 67 (17) |
|  | Bortezomib + Dexamethasone | 35 (15) | 27 (10) | 29 (8) | 28 (7) |
|  | Carfilzomib + Dexamethasone | 0 (0) | 0 (0) | 38 (11) | 39 (10) |
| Daratumumab monotherapy | Overall | 0 (0) | 0 (0) | 21 (6) | 38 (10) |
|  | Daratumumab | 0 (0) | 0 (0) | 21 (6) | 38 (10) |
| PI-IMiD-based triplets | Overall | 14 (6) | 15 (5) | 70 (20) | 77 (20) |
|  | Bortezomib + Lenalidomide + Dexamethasone | 14 (6) | 10 (4) | 13 (4) | 9 (2) |
|  | Bortezomib + Pomalidomide + Dexamethasone | 0 (0) | <5 (/) | <5 (/) | <5 (/) |
|  | Bortezomib + Thalidomide + Dexamethasone | 0 (0) | <5 (/) | <5 (/) | <5 (/) |
|  | Carfilzomib + Lenalidomide + Dexamethasone | 0 (0) | 0 (0) | 53 (15) | 43 (11) |
|  | Ixazomib + Lenalidomide + Dexamethasone | 0 (0) | 0 (0) | 0 (0) | 19 (5) |
| mAbs-based triplets | Overall | 0 (0) | 0 (0) | 14 (4) | 44 (11) |
|  | Elotuzumab + Lenalidomide + Dexamethasone | 0 (0) | 0 (0) | 13 (4) | 22 (6) |
|  | Elotuzumab + Cyclophosphamide + Dexamethasone | 0 (0) | 0 (0) | 0 (0) | <5 (/) |
|  | Daratumumab + Lenalidomide + Dexamethasone | 0 (0) | 0 (0) | 0 (0) | 8 (2) |
|  | Daratumumab + Bortezomib + Dexamethasone | 0 (0) | 0 (0) | 0 (0) | 13 (3) |
|  | Daratumumab + Cyclophosphamide + Dexamethasone | 0 (0) | 0 (0) | <5 (/) | 0 (0) |
| Other | Overall | 12 (5) | 30 (11) | 27 (8) | 15 (4) |
|  | Bortezomib + Cyclophosphamide + Dexamethasone | <5 (/) | 7 (2) | <5 (/) | <5 (/) |
|  | Bortezomib + Doxorubicin + Dexamethasone | <5 (/) | 6 (2) | 0 (0) | <5 (/) |
|  | Bortezomib + Bendamustine + Dexamethasone | <5 (/) | <5 (/) | <5 (/) | <5 (/) |
|  | Bortezomib + Melphalan + Prednison | <5 (/) | 0 (0) | <5 (/) | <5 (/) |
|  | Bortezomib + Panobinostat + Dexamethasone | 0 (0) | <5 (/) | 16 (4) | <5 (/) |
|  | Lenalidomide + Doxorubicin + Dexamethasone | <5 (/) | <5 (/) | <5 (/) | <5 (/) |
|  | Lenalidomide + Bendamustine + Dexamethasone | <5 (/) | <5 (/) | <5 (/) | 0 (0) |
|  | Thalidomide + Bendamustine + Dexamethasone | 0 (0) | <5 (/) | 0 (0) | 0 (0) |
|  | Pomalidomide + Cyclophosphamide + Dexamethasone | 0 (0) | <5 (/) | 0 (0) | 0 (0) |
|  | Pomalidomide + Bendamustine + Dexamethasone | 0 (0) | 0 (0) | 0 (0) | <5 (/) |
|  | Carfilzomib + Cyclophosphamide + Dexamethasone | 0 (0) | 0 (0) | 0 (0) | <5 (/) |
|  | Bortezomib + Pomalidomide + Doxorubicin + Dexamethasone | 0 (0) | 0 (0) | <5 (/) | 0 (0) |
|  | Bortezomib + Pomalidomide + Bendamustine + Dexamethasone | <5 (/) | <5 (/) | 0 (0) | 0 (0) |

Information for therapy regimens of less than 5 patients is not displayed for reasons of data protection.

Table S2: Definition of the study outcomes

| **Outcome** | **Definition** |
| --- | --- |
| Death | Death from any cause during the follow up. |
| Time to next therapy | Time from index date (= start of follow-up) until prescription of therapy regimen other than treatment regimen at index date during follow up. |
| Febrile neutropenia | Febrile neutropenia will be defined based on primary or secondary hospital or an ambulatory diagnosis of drug-induced neutropenia and agranulocytosis (ICD-10 GM code D70.1) in combination with a prescription of a specific antibiotic in the same quarter:   - ceftazidime (ATC codes: J01DD02, J01DD52), - imipenem (ATC code: J01DH51), - meropenem (ATC codes: J01DH02, J01DH52), - gentamicin (ATC code: J01GB03,), - amikacin (ATC codes: J01GB06, J01RA06), - vancomycin (ATC code: J01XA01), - piperacillin/tazobactam (ATC codes: J01CA12, J01CR05, J01CG02), - ceftriaxon (ATC codes: J01DD04, J01DD63, J01DD54). |
| Pneumonia | Pneumonia will be defined based on primary admission diagnosis of pneumonia (ICD-10 GM code J18) during follow up. |
| Thromobosis | Thrombosis will be defined based on primary discharge diagnosis of deep vein thrombosis (ICD-10 GM codes: I80.1, I80.2, I81, I82.2, I82.3) or pulmonary embolism (ICD-10 GM codes: I26.x) during follow up. |
| Number of red cell transfusions | Number of red cell transfusions will be defined based on coded inpatient and outpatient transfusion of erythrocyte concentrate (OPS-Code 8800.c) during follow up. |
| Number of platelet transfusions | Number of platelets transfusions will be defined based on coded inpatient and outpatient transfusion of:   - Patient-related platelet concentrates (OPS-Code 8800.6) - Pathogen inactivated apheresis platelet concentrate (OPS-Code 8800.d) - Apheresis platelet concentrate (OPS-Code 8800.f) - Platelet concentrate (OPS-Code 8800.g) - Pathogen inactivated platelet concentrate (OPS-Code 8800.h) - Other pathogen inactivated apheresis platelet concentrates (OPS-Code 8800.j) - Other platelet apheresis concentrates (OPS-Code 8800.k) - Other platelet concentrate (OPS-Code 8800.m) - Other pathogen inactivated platelet concentrates (OPS-Code 8800.n)   during follow up. |
| Number of hospitalizations | Number of hospitalizations during follow up.  Hospitalizations with at least day between discharge and admission will be considered as separate events. |
| Number of hospital days | Number of hospital days during follow up.  For each hospital stay, the number of hospital days will be calculated as (day of hospital discharge – days of hospital admission) + 1. |

**Statistical analysis.**

For time to event outcomes, i.e. time to death, time to next therapy as well as time to febrile neutropenia, pneumonia, and deep vein thrombosis, Cox proportional hazard models were fitted to compare the hazards of the event between cohorts. The proportionality of hazards assumption was examined on the basis of Schoenfeld residuals and was valid for all outcomes [1]. For count outcomes such as number of hospitalizations, number of hospital days as well as number of platelet or red cell transfusions, Poisson regression models were used to compare the rates of count outcomes between cohorts. Overdispersion was taken into account by using robust sandwich estimator for standard errors [2].

In addition, for time to event outcomes, except time to next therapy, follow up was censored at the time of treatment change, one year (end of the study period), the end of continuous enrolment, or occurrence of a specific study outcome, whichever came first. For count outcomes follow up was censored at the time of treatment change, the end of continuous enrolment, or one year, whichever came first.

The effect modification by age on the associations between cohort and outcomes was tested using interaction terms in the Cox proportional-hazards and Poisson regressions and by comparing effect estimates in the age-stratified analyses.

Data management and statistical analyses were performed using SAS 9.4 (SAS Institute Inc.) and R 3.5.2.

1. Schoenfeld D. Partial residuals for the proportional hazards regression model. Biometrika 1982;69:239–241

2. White, H. Maximum likelihood estimation of misspecifed models. Econometrica 1982;50:1–25.
